# Evolutionary process toward avian-like cephalic thermoregulation system in Theropoda elucidated based on nasal structures

**DOI:** 10.1098/rsos.220997

**Published:** 2023-04-12

**Authors:** Seishiro Tada, Takanobu Tsuihiji, Ryoko Matsumoto, Tomoya Hanai, Yasuko Iwami, Naoki Tomita, Hideaki Sato, Khishigjav Tsogtbaatar

**Affiliations:** ^1^ Department of Geology and Paleontology, National Museum of Nature and Science, 4-1-1 Amakubo, Tsukuba, Ibaraki 305-0005, Japan; ^2^ Department of Earth and Planetary Science, Graduate School of Science, The University of Tokyo, 7-3-1 Hongo, Bunkyo-ku, Tokyo 113-0033, Japan; ^3^ Department of Zoology, Kanagawa Prefectural Museum of Natural History, 499 Iryuda, Odawara, Kanagawa 250-0031, Japan; ^4^ Yamashina Institute for Ornithology, 115 Konoyama, Abiko, Chiba 270-1145, Japan; ^5^ The University Museum, The University of Tokyo, 7-3-1 Hongo, Bunkyo-ku, Tokyo 113-0033, Japan; ^6^ Institute of Paleontology and Geology, Mongolian Academy of Sciences, 15160 Ulaanbaatar, Mongolia

**Keywords:** amniota, Theropoda, *Velociraptor*, respiratory turbinate, selective brain cooling, endothermy

## Abstract

It has long been discussed whether non-avian dinosaurs were physiologically closer to ectotherms or endotherms, with the internal nasal structure called the respiratory turbinate present in extant endotherms having been regarded as an important clue for this conundrum. However, the physiological function and relevance of this structure for dinosaur physiology are still controversial. Here, we found that the size of the nasal cavity relative to the head size of extant endotherms is larger than those of extant ectotherms, with that of the dromaeosaurid *Velociraptor* being below the extant endotherms level. The result suggests that a large nasal cavity accommodating a well-developed respiratory turbinate is primarily important as a thermoregulation apparatus for large brains characteristic of endothermic birds and mammals, and the nasal cavity of *Velociraptor* was apparently not large enough to carry out this role required for an endothermic-sized brain. In addition, a hypothesis that the enlargement of the nasal cavity for brain cooling has been associated with the skull modification in the theropod lineage toward modern birds is proposed herein. In particular, the reduction of the maxilla in derived avialans may have coincided with acquisition of the avian-like cephalic thermoregulation system.

## 1. Introduction

In dinosaur palaeontology, one of the major interests has been their physiology, especially concerning the question ‘Were dinosaurs endothermic or ectothermic?’ (e.g. [1]). Endothermic animals can maintain a high body temperature by internal heat sources whereas ectothermic animals depend on external heat sources, primarily solar radiation, to keep the body at an appropriate temperature [2]. In general, birds and mammals are endotherms whereas lepidosaurians, testudines and crocodylians are ectotherms among extant amniotes [3], although there are some exceptions (e.g. [4]). Because non-avian dinosaurs are phylogenetically positioned between plesiomorphic, ectothermic animals and apomorphic, endothermic birds [5], it is generally assumed that endothermy must have been achieved along the lineage toward Aves (e.g. [3]; note, however, there is another hypothesis postulating that the most recent common ancestor of Archosauria was endothermic, with extant crocodylians representing a secondary reversal to ectothermic animals (e.g. [6,7])). However, no consensus has been reached regarding the timing of acquisition of endothermy or the metabolic modes of non-avian dinosaurs (e.g. [6,8–14]). To shed new light on this issue, a more rigorous approach based on an objective proxy is necessary. The nasal structure has been considered such a proxy of the metabolic status of extinct dinosaurs.

**Figure 1.**
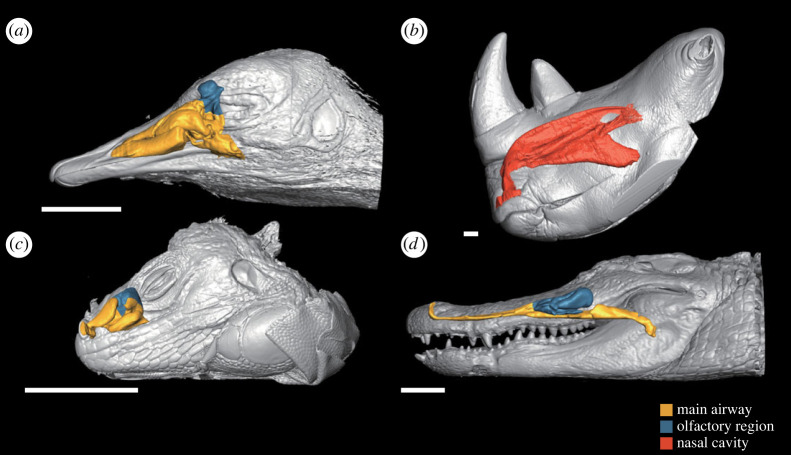
Three-dimensional reconstructions of the nasal cavities of extant endotherms (*a*,*b*) and ectotherms (*c*,*d*) based on CT scan data. (*a*) *Struthio camelus* (Aves; OUVC 10491), (*b*) *Ceratotherium simum* (Mammalia; OUVC 9754), (*c*) *Iguana iguana* (Lepidosauria) and (*d*) *Alligator mississippiensis* (Crocodylia; OUVC 9761). Scale bars equal to 5 cm.

The nasal cavities of birds and mammals accommodate structures called respiratory turbinates unique to these animals among extant taxa (figure 1). The respiratory turbinate is a complex structure protruding into the nasal cavity and generally scroll-like in shape in birds [15]. Because this complex structure increases the surface area of the nasal cavity, it is considered enabling efficient heat and water exchanges between the mucosa and inhaled and exhaled air, thus compensating for extra heat and water losses caused by efficient lung ventilation typical of endotherms [16,17]. In addition, birds and mammals, which achieved endothermy independently from each other, also acquired the respiratory turbinate convergently [18]. Therefore, the association between endothermy and the presence of the respiratory turbinate appears fairly tight, making these structures potentially informative in evaluating the metabolic status of amniote animals, especially fossil forms [19–22]. Although the generally cartilaginous nasal turbinate itself is rarely completely preserved in the fossil record in the lineage toward birds because of its fragileness [23,24], the size of the nasal cavity, which may be expected to be larger in endotherms for accommodating this structure, may offer a clue for the metabolic status of fossil forms. Ruben *et al.* [11] conducted a seminal study focusing on this characteristic for inferring the dinosaur metabolic mode. However, there are a couple of issues in the analytical method used in their study, as have been pointed out previously. Firstly, the nasal cross-sectional area used for the parameter of the nasal cavity size in Ruben *et al.* [11] varies dramatically throughout a single nasal cavity [23,25]. Secondly, the body mass, against which the cross-sectional area of the nasal cavity was regressed in Ruben *et al.* [11] for comparison between endotherms and ectotherms, is subject to serious errors in estimation or measurement not only for non-avian dinosaurs, but also for extant animals [25,26]. Therefore, the links between the respiratory turbinate and endothermy still remain controversial [27].

Selective brain cooling has been suggested more recently as another physiological function of the respiratory turbinate [28,29]. In fact, the nasal region as a whole has been interpreted as one of the most important body parts for heat exchange in cooling the brain [29–34]. However, the respiratory turbinate is also considered playing only a minor thermophysiological role in birds by some authors (e.g. [28]). Thus, the function of the respiratory turbinate in extant amniotes and its implication for the physiology of fossil species are still worth for exploring.

As an attempt of solving these issues, the present study first clarifies whether or not the volume and surface area of the nasal cavity, which are expected to be correlated with the presence and absence of the turbinate, are quantitatively different between extant ectotherms and endotherms. Then, palaeontological applications of the results obtained on extant taxa are discussed using the dromaeosaurid theropod *Velociraptor mongoliensis* as an example. Finally, by combining the data on extant taxa and the fossil record of Theropoda, a hypothesis that the enlargement of the nasal cavity facilitated brain cooling and was associated with the skull modification in the theropod lineage toward modern birds is proposed.

### 1.1. Institutional abbreviations

CMNH, Cleveland Museum of Natural History, Cleveland, OH, USA; MPC-D, Institute of Paleontology and Geology, Mongolian Academy of Sciences, Ulaanbaatar, Mongolia; OUVC, Ohio University Vertebrate Collections, Athens, OH, USA.

## 2. Material and methods

The nasal cavity of a wide range of extant amniotes was three-dimensionally reconstructed based on X-ray computed tomography (CT) scan data of head specimens in order to obtain measurements of the surface area and volume for comparison between endotherms and ectotherms. In addition, the reconstructed nasal cavity in each specimen was divided into the regions for respiration and olfaction and the surface area and volume of the former only was further compared between these forms. For each comparison, apparent differences between endotherms and ectotherms were then tested by multiple statistical methods. Furthermore, the nasal cavity of a non-avian dinosaur *V. mongoliensis* was reconstructed based on CT-scan data of the skull by taking spatial osteological constraints into consideration. Measurements were then made on the reconstructed nasal cavity.

### 2.1. Computed tomography scanning, segmentation and measurement

Head specimens of extant amniotes were CT-scanned for reconstructing the three-dimensional nasal cavities through digital segmentation. In addition, CT datasets were taken from openly available datasets (MorphoSource, DigiMorph, Yamashina Institute for Ornithology Specimen Database and Digital Morphology Museum). In total, 51 specimens of 47 species were included for the present analyses: 21 species in Aves, eight in Mammalia, four in Crocodylia, three in Testudines and 11 in Lepidosauria (table 1). Among these clades, avian data were collected phylogenetically most comprehensively because the present study mainly focused on physiological changes having occurred across the non-avian dinosaur-avian transition. Individuals of different ontogenetic stages of *Struthio camelus* and *Larus crassirostris* (Aves), as well as *Alligator mississippiensis* (Crocodylia), were also examined in order to discuss whether there is a difference between ontogenetic and interspecific allometry. Detailed information of specimens and CT-scanning conditions are provided in electronic supplementary material, table S1.

**Table 1.** Taxa examined in this study. Asterisks show the species of which multiple specimens were used. Detailed information of the examined specimens is summarized in electronic supplementary material, table S1.

**Endotherms**	**Ectotherms**
**Aves**	**Crocodylia**
Struthio camelus*	*Crocodylus porosus*
*Dromaius novaehollandiae*	*Crocodylus siamensis*
*Coturnix japonica*	Alligator mississippiensis*
*Numida meleagris*	*Caiman crocodilus*
*Anas platyrhynchos*	**Testudines**
*Chordeiles minor*	*Chelodina mccordi*
*Musophaga violacea*	*Chelydra serpentina*
*Columba livia*	*Geoclemys hamiltonii*
*Gallinula chloropus*	**Lepidosauria**
*Phoenicopterus roseus*	*Nephrurus amyae*
*Fratercula cirrhata*	*Eumeces schneideri*
*Fratercula arctica*	*Heloderma suspectum*
Larus crassirostris*	*Pseudopus apodus*
*Gavia immer*	*Varanus albigularis*
*Phoebastria nigripes*	*Varanus exanthematicus*
*Phalacrocorax carbo*	*Chlamydosaurus kingii*
*Alcedo atthis*	*Furcifer pardalis*
*Sarcoramphus papa*	*Iguana iguana*
*Loriculus galgulus*	*Physignathus concincinus*
*Corvus macrorhynchos*	*Python regius*
*Passer montanus*	
**Mammalia**	**Dinosauria**
*Gorilla gorilla*	*Velociraptor mongoliensis*
*Macaca fuscata*	
*Pan troglodytes*	
*Symphalangus syndactylus*	
*Panthera leo*	
*Tapirus indicus*	
*Equus quagga*	
*Ceratotherium simum*	

Based on each CT-scan dataset, the nasal cavity was segmented three-dimensionally using the software Amira v. 6.0.1 and 6.2.0 (FEI, OR, USA) and 2021.2 (Thermo Fisher Scientific, TX, USA; figure 1). The reconstructed nasal cavity of several birds, crocodylians and lepidosaurians was further divided into regions for respiration and olfaction for comparison of the former region only. The boundary between these regions was drawn based on the distribution of the olfactory and respiratory epithelia described in the past literature [15,23,35,36]. With some exceptions [15], the region lined with the olfactory epithelium in birds and crocodylians generally accommodates corresponding structures, i.e. olfactory turbinate in Aves and concha and postconcha in Crocodylia [15,23]. Although the anterior part of the nasal passage, nasal vestibule, is not lined with the respiratory epithelium [37], this part was assigned to the main airway together with the nasal cavity proper in the present study because it contributes to the course for the main flow of inhaled and exhaled air [38]. In Lepidosauria, on the other hand, the nasal cavity varies in size and shape drastically and shows no universal pattern of the epithelial distribution [36]. Accordingly, the regional division was done only for lepidosaurian taxa described in Gabe & Saint-Girons [36]. Division of these two regions was not attempted in mammals because they have more complex turbinates than any other amniotes [39] and no clear structural landmarks are available for drawing the boundary between the respiratory and olfactory regions. Accordingly, data on mammals were not included in the analysis on the respiratory region only.

In addition to the nasal cavity, the skull (i.e. the upper and lower jaws, excluding the sclerotic rings and hyoid bones) was reconstructed based on the same dataset. For each specimen, the surface area (mm^2^) and volume (mm^3^) of the nasal cavity, as well as the volume (mm^3^) of bones comprising the skull, were then measured in Amira. The volume of the skull was used as a parameter representing the volume of the head readily available on fossil specimens lacking associated soft tissues such as muscles, volumetric reconstruction of which would involve much uncertainty and some degree of speculation. For a better approximation of the head volume requiring the fewest assumptions, however, the minimum convex hull of each skull was also created using the software MeshLab v. 2022.02 (Visual Computing Lab, ISTI-CNR, Pisa, Italy). Because the convex hull model includes all the vertexes on the skull minimally and fills foramina and cavities in the skull based on a single criterion, it is an appropriate proxy for the head size that can be created without assumptions concerning the volumes of soft tissues. The volume of this head model was also measured and used in the following analyses.

### 2.2. Comparisons and statistical analyses on extant amniotes

The size of the nasal cavity and its respiratory region represented by the surface area and volume were regressed against the body mass (g), skull volume (mm^3^) and volume of the convex-hulled skull (mm^3^) for comparison between ectotherms and endotherms. The body mass represents their body size, whereas the volume of the skull and the convex hull model reflect their head size. Many specimens lacked data of the body mass except for a few specimens for which it was directly measured. For specimens lacking such data, the body mass data were assigned in the following ways. For birds, the mean value of each species listed in Dunning [40] was used. To verify the validity of using such data, the body mass directly measured on the specimens and the mean value for the corresponding species listed in Dunning [40] were compared and they were largely consistent with each other. For crocodylians, the body mass was calculated based on allometric equations provided by Webb & Messel [41] and Farlow *et al.* [42].

Raw data were logarithmically transformed before calculating phylogenetic generalized least-squared regression (PGLS), reduced major axis regression (RMA) and ordinary least-squared regression (OLS) equations for endotherms and ectotherms. All data analyses were carried out with the statistical program R v. 4.0.5 [43]. OLS, RMA and PGLS fittings were conducted by the R packages (OLS, {stats} [43]; RMA, {smatr} [44]; PGLS, {ape} [45] and {nlme} [46,47]).

Statistical significance of the difference in the RMA, OLS and PGLS allometric lines between endothermic and ectothermic animals was tested in the following ways. Firstly, the difference in the slopes of allometric lines between these animals was tested for each regression analysis. Secondly, when no statistically significant difference was found in the slopes, the difference in the intercepts between those lines was examined through analysis of covariance (ANCOVA). The R packages {stats} and {smatr} were used for ANCOVA on the OLS and RMA regression lines, respectively, and {ape} and {nlme} for phylogenetic ANCOVA [48] on the PGLS regression lines.

For PGLS fitting, a phylogenetic tree containing all examined species with information on branch length was constructed using TIMETREE (timetree.org; [49]). Because *Chelodina longicollis* was not included in the dataset of TIMETREE, the data of the closely related *Chelodina mccordi* was used instead. Subtrees used for each analysis were produced by culling taxa from this tree. PGLS analysis accommodates only one operational taxonomic unit (OTU) for each species. For a species including data of multiple specimens, the branch length of 1 Ma was assigned for each individual belonging to the single species manually in the present analysis.

In addition, this study made a preliminary attempt at elucidating the relationship between the size of the nasal cavity and metabolic rate. The method and result are described in the electronic supplementary material.

### 2.3. Reconstruction of the nasal cavity in the dromaeosaurid *Velociraptor*

For comparison with data on extant amniotes, the nasal cavity of a possible maximum size was three-dimensionally reconstructed in the dromaeosaurid *V. mongoliensis* (MPC-D 100/2000) based on its CT-scan dataset. Because the skull of this specimen suffers from relatively little deformation, it was suitable for the present purpose. The scanning parameters are provided in electronic supplementary material, table S1. Osteological features related to soft tissues and positions of soft structures reconstructed in past studies were used for spatial constraints. They are the fleshy nostril position [50], course of the nasal passage based on the inner side of the maxilla [25,51], choanal position [29] and caudal end of the nasal cavity indicated by the position of the mesethmoid [52]. Segmentation of the skull was conducted in the same way as was done for extant animals described above. Since only the right postdentary bones were lacking, the volume of these bones on the left side was added to that of the preserved part to obtain the volume of the whole skull. The convex hull of the skull was also produced.

**Figure 2.**
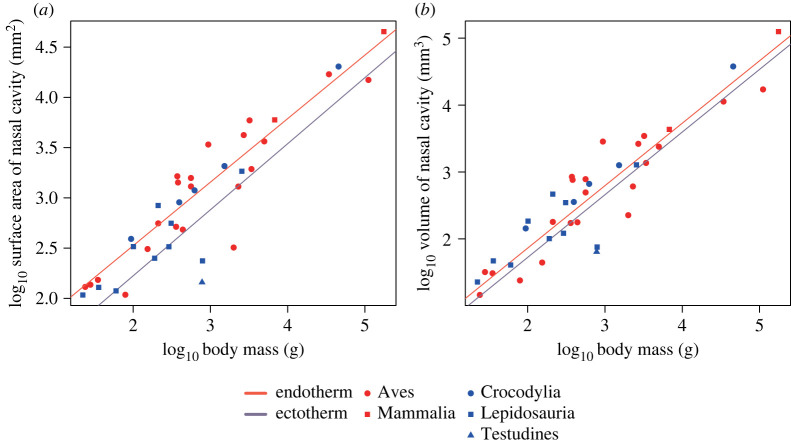
Relationships between the nasal cavity size and body mass. The size of the nasal cavity is represented by the surface area (*a*) and volume (*b*), respectively. The regression lines were PGLS-fitted.

**Figure 3.**
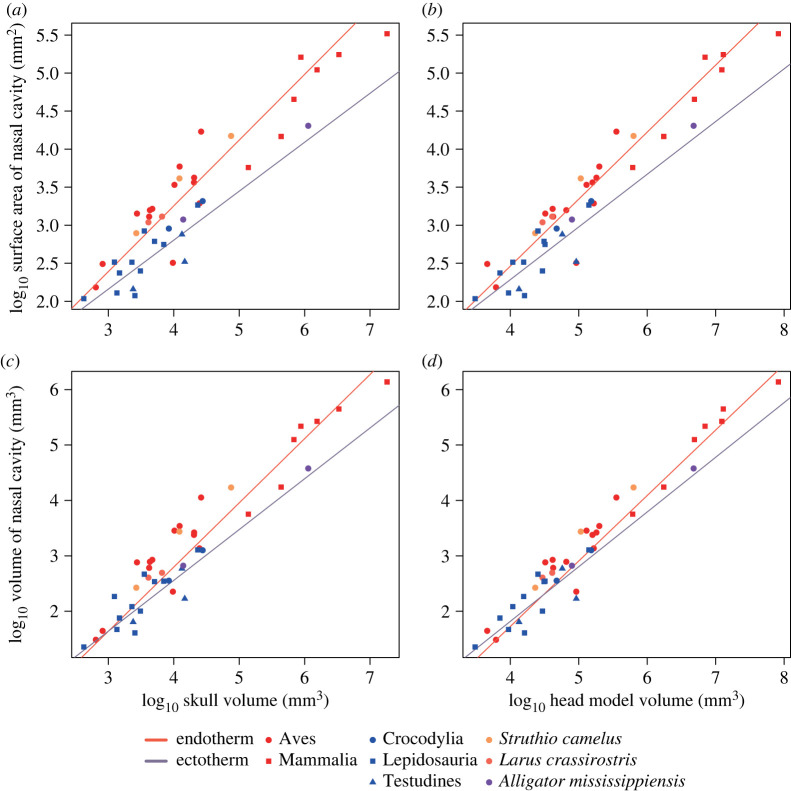
Relationships between the nasal cavity size and the skull volume (*a*,*c*) and head model volume (*b*,*d*). The size of the nasal cavity is represented by the surface area (*a*,*b*) and volume (*c*,*d*), respectively. The regression lines were PGLS-fitted.

**Figure 4.**
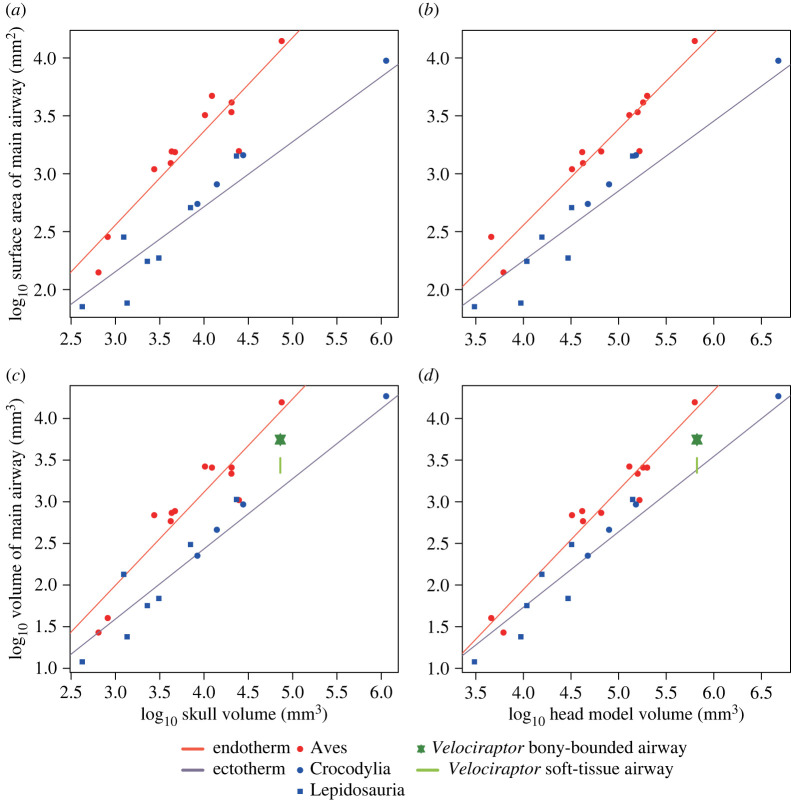
Relationships between the respiratory region of the nasal cavity and the skull volume (*a*,*c*) and head model volume (*b*,*d*). The size of the nasal cavity is represented by the surface area (*a*,*b*) and volume (*c*,*d*), respectively. The regression lines were PGLS-fitted. The green lines show the 40–60% range of the volume of the maximum main airway of *Velociraptor mongoliensis* (MPC-D 100/2000).

The volume of the reconstructed nasal cavity was measured for comparison with extant amniotes. The volume, not the surface area, was used because the surface area of the nasal cavity in an animal drastically changes dependent on whether or not the turbinate is included for measurement. In addition, a complete respiratory turbinate is rarely preserved in fossils belonging to the reptilian lineage because it is very fragile and the avian turbinate is also often made of cartilage only, unlike ossified ones in mammals (e.g. [23,24,53]). By contrast, the volume of the nasal cavity can be compared independent of the preservation condition of the turbinate. Furthermore, the presence of the turbinate may be expected to expand the nasal cavity and could be reflected by the volume of the latter, possibly making an indirect inference of its presence in fossils based on this parameter possible. Accordingly, the volume is herein considered most appropriate for comparison of the size of the nasal cavity across extant and fossil species. It was also confirmed that the relationship between the regression lines of endotherms and ectotherms in each analysis shown below remained generally the same when the volume was used instead of the surface area (figures 2–4).

## 3. Results

Raw measurements including those collected from literature are summarized in electronic supplementary material, table S2. Although three regression lines, PGLS, RMA and OLS, were calculated, only PGLS regression lines were shown in the following figures because all three regression lines show similar trends. Scatter diagrams with all regression lines were provided in electronic supplementary material, figure S1, and the results of all statistical analyses were provided in electronic supplementary material, table S3.

### 3.1. Relationships between the nasal cavity size and body mass

The relationships between the body mass and size parameters (surface area and volume) of the nasal cavity are shown in figure 2*a*,*b*. The size parameters of the nasal cavity were positively correlated with the body mass both in endotherms and ectotherms. The correlation coefficients of the regression lines were all high (electronic supplementary material, table S3). The regression lines for endotherms and ectotherms were similar to each other, especially for the nasal cavity volume. In fact, as shown in table 2 and electronic supplementary material, table S3, there was no statistically significant difference in the slopes or intercepts found in any comparisons.

**Table 2.** Results of statistical tests on the difference in the PGLS allometric lines between endotherms and ectotherms. *p*-values in Italics indiciate a statistically significant difference at *p* = 0.05. Detailed results of statistical analyses are shown in electronic supplementary material, table S3.

	nasal cavity versus body mass		nasal cavity versus skull volume		nasal cavity versus head model volume		respiratory region versus skull volume		respiratory region versus head model volume	
	slope	intercept	slope	intercept	slope	intercept	slope	intercept	slope	intercept
*surface area*										
*p*-value	0.70	0.50	<*0.001*	—	<*0.001*	—	*0.023*	—	*0.014*	—
*volume*										
*p*-value	0.97	0.81	<*0.001*	—	<*0.001*	—	0.090	<*0.001*	*0.015*	—

### 3.2. Relationships between the nasal cavity and head sizes

In the analyses on the nasal cavity size relative to the head size, the relationship of the regression lines between endotherms and ectotherms was generally the same regardless of whether the volume of the skull or the convex-hulled head model was used as a parameter of the head size.

#### 3.2.1. Whole nasal cavity size relative to the volume of the skull and head model

The relationships between the volume of the skull or the head model and size parameters of the nasal cavity are shown in figure 3*a–d*. Both surface area and volume of the nasal cavity were positively correlated with the skull volume or the volume of the head model both in endotherms and ectotherms. The correlation coefficients of the obtained regression lines were all high (electronic supplementary material, table S3). Contrary to the analyses using the body mass, a significant difference was found either in the slopes or intercepts between endotherms and ectotherms (table 2).

#### 3.2.2. The size of the respiratory region in the nasal cavity relative to the volume of the skull and head model

The relationships between the volume of the skull or the head model and size parameters of the respiratory region in the nasal cavity are shown in figure 4*a–d*. The correlation coefficients were high for all regressions (electronic supplementary material, table S3). Although both size parameters of the respiratory region were correlated with the volume of the skull or the head model positively both in endotherms and ectotherms, the regression lines differed greatly between them, with a significant difference being found either in the slopes or intercepts in all comparisons (table 2, electronic supplementary material, table S3), suggesting that allometry of the size of the respiratory region of the nasal cavity relative to the volumes of both skull and head model is different between the two metabolic modes in diapsids.

### 3.3. Evaluation of the physiological function of the nasal cavity in non-avian dinosaurs

#### 3.3.1. Reconstruction of the nasal cavity in non-avian theropod dinosaurs

The actual nasal cavity bounded by soft tissues in a fossil form would have occupied only a portion of the empty space surrounded by the facial and palatal bones in the skull, i.e. the maximum nasal cavity or bony-bounded nasal cavity [23,29]. Accordingly, for inferring or reconstructing the size and shape of the original nasal cavity, the maximum nasal cavity needs to be further constrained spatially using anatomical landmarks. In the present study, the following constraints were applied to reconstruction of the nasal cavity in non-avian theropods:

I. The fleshy nostril being located on the rostralmost part of the bony nostril [50].
II. The inner structures of the maxilla restricting the main nasal passage to the upper part of the internal skull [25,51]: the maxillary antrum wall [54] and preantral and postantral struts [55,56] are the inner structures that are profoundly correlated with the maxillary antrum, which would have held a part of the paranasal air sinuses [51,55].
III. The fleshy choana being positioned on the caudal edge of the bony choana [29]: in birds, the choana is associated with a depression on the palatine referred to as the choanal fossa [57]. A similar structure can be seen on the same bones of non-avian dinosaurs. In addition, this structure constrains the course of the nasopharyngeal duct to some extent. That is, a truly vertical direction would be impossible for the nasopharyngeal duct. Furthermore, most of the extant diapsids observed herein had their olfactory region located caudal to the position of the fleshy choana and the main airway not extending more posteriorly than the latter (figure 1), suggesting that this condition was also probably applicable to non-avian theropod dinosaurs.
IV. The caudodorsal part of the nasal cavity, i.e. olfactory cavity, reaching the rostral terminus of the median septum of the mesethmoid (or a dorsally extending parasphenoid as exceptionally present in *Allosaurus*; [52]): the median septum of the mesethmoid structure supports the olfactory nerves leading forward to the nasal cavity. Therefore, its rostral ends attach to the caudodorsal end of the nasal cavity [52].

**Figure 5.**
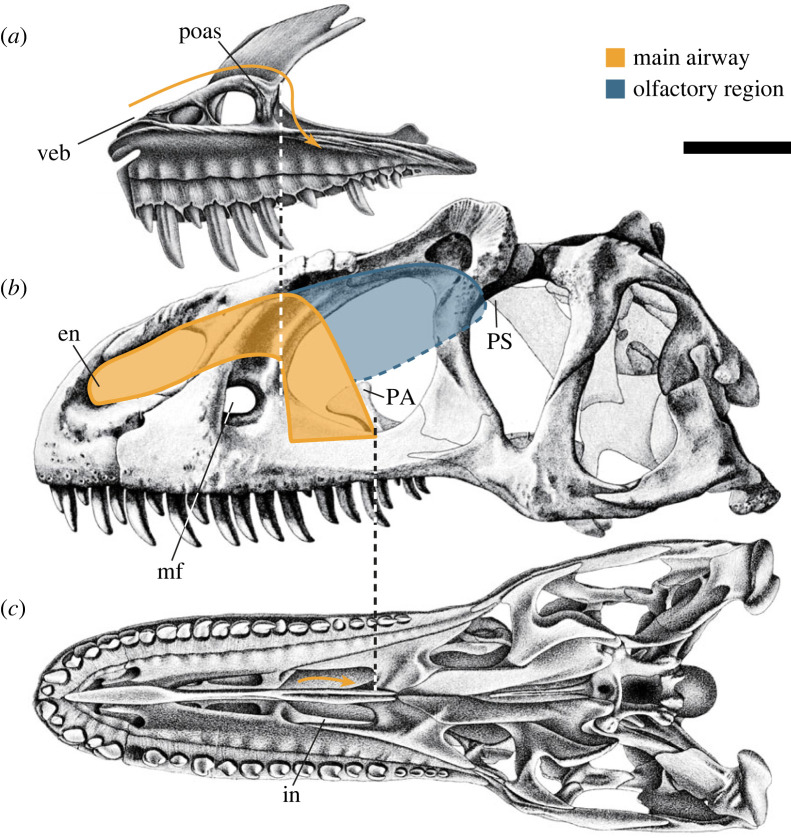
Diagram showing the present scheme of reconstruction of the possible nasal cavity in a theropod dinosaur based on osteological constraints, as exemplified by *Allosaurus fragilis*. The maxilla in medial view (*a*). The skull in left lateral (*b*) and ventral views (*c*). Dashed line indicates ambiguous limits of the nasal cavity. Scale bar equals to 10 cm. en, external naris; in, internal naris/choana; mf, maxillary fenestra; PA, palatine; poas, postantral strut; PS, parasphenoid; veb, vestibular bulla. The base illustration modified from Madsen [59] with permission from the Utah Geological Survey.

Although other soft-tissue factors such as mucosal constrictions [58] were not taken into consideration herein, hypothetical nasal cavities were schematically reconstructed in theropod dinosaurs. The shape of the reconstructed nasal cavity constrained was tube-like and consistent across non-avian theropods (figure 5).

#### 3.3.2. The physiological function of the nasal cavity of *Velociraptor mongoliensis*

**Figure 6.**
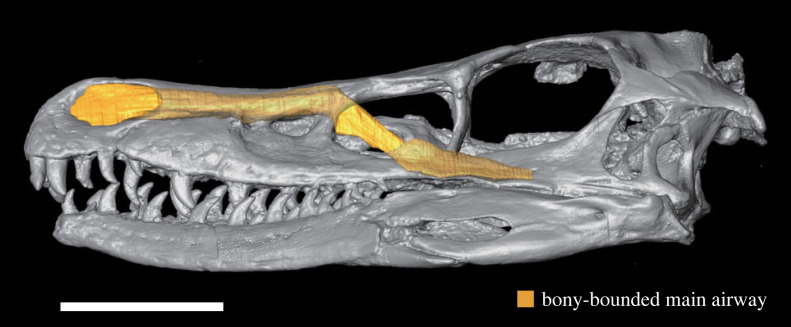
Three-dimensional reconstruction of a possible respiratory region of the nasal cavity in *Velociraptor mongoliensis* (MPC-D 100/2000). Scale bar equals to 5 cm.

By applying the above criteria, the main airway of *V. mongoliensis* (MPC-D 100/2000) was reconstructed three-dimensionally (figure 6). The size of the region was compared by plotting it with data of extant diapsids on the scatter diagram showing the relationship between the volume of the skull or the head model and volume of the respiratory region of the nasal cavity. As a result, *V. mongoliensis* was plotted at an almost exact midpoint between regression lines of endotherms and ectotherms (figure 4*c*,*d*). Because soft-tissue airways comprise approximately 40–60% of the bony-bounded nasal passage in diapsids [58], the actual nasal airway of *V. mongoliensis* would be located more downward in this plot (figure 4*c*,*d*). No osteological correlate of the respiratory turbinate was identified in this specimen [24].

## 4. Discussion

### 4.1. Primary function of the nasal turbinate

When regressed against the body mass, the size of the nasal cavity represented by the surface area and volume shows no significant difference between endotherms and ectotherms (figure 2). Rather, the nasal cavity appears to have a universal relationship with the body mass in extant amniotes. This result is in contrast to the hypothesis proposed by Ruben *et al.* [11] that the size of the nasal cavity relative to the body mass is different between endotherms and ectotherms. The present result instead showed that the sizes of the nasal cavity and its respiratory region, represented both by the surface area and volume, were different between endotherms and ectotherms when regressed against the skull volume and the volume of the convex hull, with endotherms tending to have a larger size (figures 3 and 4). These results suggest that the respiratory turbinate, of which presence is reflected by a large-sized nasal cavity, has a role primarily affecting the head region and not the whole body. We herein support a hypothesis that the primary function of this structure is heat exchange necessary for a large brain typically present in birds and mammals [28]. The nasal cavity has been interpreted as one of the most important regions for heat exchange in cooling the brain [29–34]. In diapsids, numerous branches of the nasal arteries extend along the nasal cavity and then merge into major venous pathways toward the brain or the retina when present, serving for selective brain cooling [29,31–34], as is also the case with mammals [60,61]. Their nasal cavity possessing the respiratory turbinate functions more efficiently for their larger brains requiring more extensive thermal exchange [62–65].

Owerkowicz *et al.* [28] concluded that the respiratory turbinate of birds plays only a minor thermophysiological role compared with the one in mammals considering their smaller surface area of the respiratory turbinate. However, because the distribution of vessels in the nasal region contributing to heat exchange is not limited to the surface of the respiratory turbinate and extends to other surfaces of the nasal cavity [33,66,67], the rationale and conclusion of Owerkowicz *et al.* [28] may not be totally justified. Although the other cranial regions, for example, the orbital region holding the ophthalmic rete, may contribute to the control of brain temperature more efficiently in birds (e.g. [28,68]), the nasal cavity and respiratory turbinate still probably function as one of the main heat exchangers for selective brain cooling in birds [33].

The hypothesis that the respiratory turbinate primarily works in selective brain cooling does not renounce the classic concept that the respiratory turbinate is beneficial in reducing the otherwise dramatically accelerated rates of evaporative heat and water loss that accompanies high respiratory rates typical of endothermic taxa [19,69,70]. In fact, these two functions are indeed based on the same phenomenon that inhaled air is heated in the nasal passage by taking heat away from warm blood distributed beneath the mucosa. In this process, the nasal passage serves as a heat sink for hot arterial blood coming from the body core [29]. At the same time, venous blood flowing from there toward the brain is cooled down. However, the nasal turbinate is apparently not involved in a physiological mechanism contributing to the whole body as suggested by the present results that the size of the nasal cavity, which is probably correlated with the presence/absence of the turbinate, was not significantly different between ectotherms and endotherms when it was regressed against the body mass. In addition, the nasal cavity size normalized by the skull volume and the volume of the convex hull head model were found hardly correlated with mass-specific basal metabolic rate (BMR), with a quite low correlation coefficient (see electronic supplementary material). These results are consistent with the hypothesis proposed by Owerkowicz *et al.* [28] that heat and water conservation at the turbinate seen in birds may be an exaptation of their original role in selective brain cooling.

The high amount of dispersion around each regression line of the size of the nasal cavity against the skull volume and the volume of the head model for endotherms and ectotherms might be partly explained by the difference in their ecology. For example, primates (*Gorilla gorilla*, *Macaca fuscata*, *Pan troglodytes* and *Symphalangus syndactylus* in this study) have a nasal cavity much reduced in size, with the internal architecture having been radically modified because their olfaction has become of reduced importance [39]. In addition, phalacrocoracid birds (*Phalacrocorax carbo* in the present study) have a greatly reduced nasal cavity because they chase their food visually and do not depend on olfaction [15]. Thus, it is here proposed that the variation in the size of the nasal cavity observed among extant taxa is mainly due to their degrees of reliance on olfaction, rather than environmental conditions of their habitats [70]. In addition, interspecific and ontogenetic allometry of select species were mostly indistinguishable from each other both in endotherms and ectotherms, suggesting that inclusion of non-adult specimens would not cause a significant problem in these allometric analyses on the nasal cavity size.

### 4.2. The sizes of the nasal cavity and brain of *Velociraptor* and other non-avialan dinosaurs

The size of the reconstructed bony-bounded nasal cavity of *V. mongoliensis* compared with those of extant endotherms and ectotherms (figure 4*c*,*d*) indicates that this dinosaur was unlikely to have a brain-cooling capability seen in modern birds. Because *V. mongoliensis* is among the most derived non-avialan theropods, most other non-avialan theropod dinosaurs may also not have possessed a large nasal cavity that accommodated a turbinate developed enough to cool a large brain. This hypothesis agrees with some studies [64,71,72] inferring that the relative sizes of brains in non-avialan dinosaurs were not as large as those in birds. The results of these studies are highly relevant because the hypothesis presented herein postulates that the brain size was the crucial factor in developing the respiratory turbinate and, in turn, acquiring a large nasal cavity. This hypothesis suggests that non-avialan dinosaurs thrived without a large-sized nasal cavity because their brains were not as fully developed as those of birds and did not require as much thermal exchange. On the other hand, the size of the nasal cavity would be irrelevant to the discussion on the metabolic status of *V. mongoliensis* if the present hypothesis holds true.

Although no osteological correlate of the respiratory turbinate was identified based on the CT-scan data of the specimen, it does not necessarily indicate that this structure was lacking given that the cartilaginous turbinates in most birds also do not leave an osteological correlate [24]. At present, only one non-avian theropod specimen, CMNH 7541 (either a juvenile *Tyrannosaurus* or distinct *Nanotyrannus*), has been reported to possess a possible respiratory turbinate [73]. CT-scan data of this specimen shows the apparently scroll-like shape of this structure, which might be applicable to that of *V. mongoliensis,* if it was indeed present. Although the scroll-shape of the turbinate has been considered playing a crucial role in increasing the efficiency of heat exchange in endotherms [11,22], that of the non-avian theropod dinosaurs, if present, would not have been developed enough to cause expansion of the nasal cavity, unlike in modern birds. The size of the nasal cavity of *V. mongoliensis* supports this hypothesis.

Although this study employed reliable constraints currently available for the reconstruction of the dinosaur nasal cavity, more anatomical information on the nasal region both in extant taxa and fossil dinosaurs is still warranted, especially concerning the olfactory region of non-avialan theropods and the whole nasal cavity of non-theropod dinosaurs. Quantitative estimates on these sizes based on more refined criteria and their comparison with the dataset of extant taxa obtained in the present study would shed new light on the comprehensive dinosaur thermoregulation strategy.

### 4.3. Evolutionary process toward avian-like cephalic thermoregulation system in Theropoda elucidated based on nasal structures

**Figure 7.**
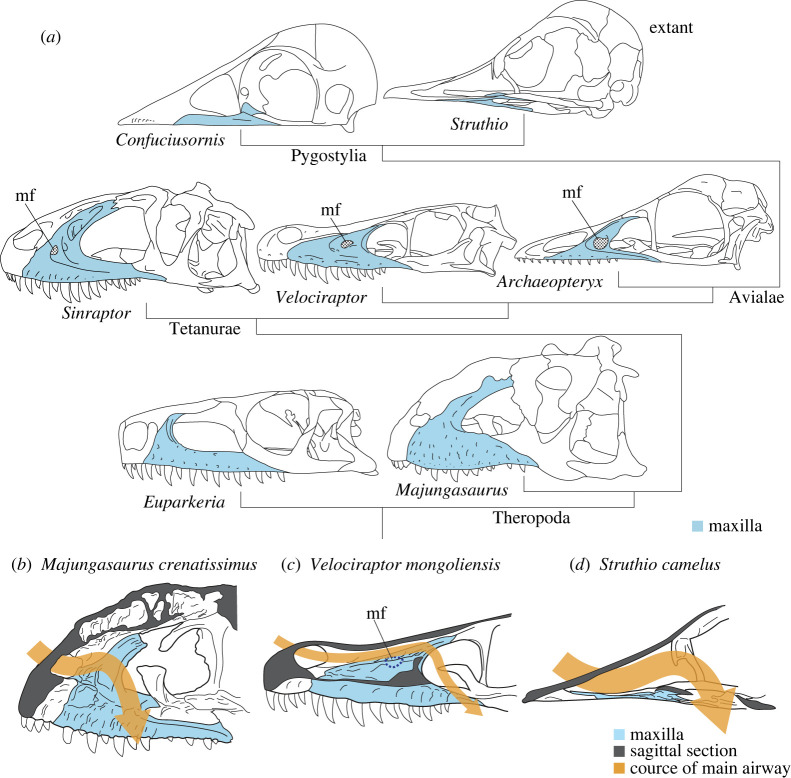
Transformation of the facial skeleton in Theropoda in lateral views (*a*), and right side of the skulls of *Majungasaurus crenatissimus* (*b*), *Velociraptor mongoliensis* (*c*) and *Struthio camelus* (*d*) in medial views. The maxilla has been greatly modified throughout the evolution toward extant birds including changes in its internal structures that affect the morphology of the nasal pathway. Skull drawings modified from Currie & Zhao [74], Barsbold & Osmólska [75], Paul [25], Sampson & Witmer [76], Elzanowski *et al.* [77], Sookias *et al.* [78] and original specimens. mf, maxillary fenestra.

Based on the nasal structures, some clues for evolutionary transformation of the skull and, albeit indirectly, the timing of acquisition of avian-like cephalic thermoregulation system can be obtained (figure 7). As the spatial constraints used for reconstructing the possible main airway of the nasal cavity above show (figure 5), the maxilla has a major influence on the shape of the nasal passage in theropod dinosaurs [22]. In addition, the large and posteriorly placed maxillary fenestra is a conspicuous landmark for the course of the nasal pathway (mf; figures 5 and 7) because it shows the presence of the maxillary antrum housing the maxillary sinus, a diverticulum of the paranasal air sinus [51,55].

Since there was no large maxillary fenestra on the maxilla in non-tetanuran theropod dinosaurs [5], it is unlikely that they possessed a developed maxillary sinus (figure 7) [51]. By contrast, the presence of a prominent maxillary fenestra in tetanurans suggests that they had a well-developed sinus in the maxillary antrum (figures 5–7) [54,55]. On the lineage toward modern birds, the maxilla was compressed downward and eventually reduced into a flat, predominantly palatal element [53,79]. During this process, the maxillary fenestra disappeared, indicating that the paranasal sinus moved more caudally as in modern birds (figure 7) [51].

This modification of the maxilla and accompanied transformation of the paranasal sinuses indicate possible changes in the morphology of the nasal pathway (figure 7). In basal theropods, the pathway of the nasal cavity from the external naris to the choana must have been wide but relatively short and simple compared with those in early diverging tetanurans (figure 7*b*). The typical non-avialan tetanurans in which the maxillary fenestra housed the sinuses are likely to have had a tube-like nasal pathway (figure 7*c*). As shown in the reconstruction of *Velociraptor*, the nasal cavity at this stage was still not large enough to regulate heat for an avian-like, large brain. In more derived avialans, the drastic reduction of the maxilla allowed the nasal cavity to enlarge in cross-section. This is the feature of the cavity in extant birds most distinct from those in non-avialan theropod dinosaurs [11] (figures 1*a* and 7*d*), and the nasal cavity at this stage may have finally been capable of cooling down a large brain typical of extant birds. The loss of the maxillary fenestra appears to represent a ‘diagnostic’ feature of acquisition of an avian-like cephalic thermoregulation apparatus (figure 7*a*). It is herein tentatively proposed that this condition had been achieved around the common ancestor of Pygostylia [80], given that this clade includes *Confuciusornis* [77] and *Yangavis* [81], both of which had reduced the maxillary fenestra, and *Cratonavis* [82], which had an unreduced triangular maxilla bearing the maxillary fenestra, although the homology of the latter structure is sometimes questioned [83]. Based on this criterion, *Archaeopteryx* would have possessed a condition rather similar to that of non-avialan theropod dinosaurs, and this hypothesis is consistent with the observation that the brain size of *Archaeopteryx* was not nearly as large as in extant birds (figure 7*a*) [71,72].

Compared with modern birds and crocodylians, non-avialan theropod dinosaurs had greatly developed antorbital sinuses in their antorbital cavity [55]. It is interpreted as a unique site of heat exchange that represents a part of a novel thermoregulatory strategy in theropods [34] and has been greatly reduced later in modern birds. This skull transformation may, therefore, indicate that the change in the prominent region for thermal exchange from the antorbital cavity to the nasal cavity within the limited space of the cranium. It may be reasonable to postulate that the nasal cavity equipped with a complex respiratory turbinate is more efficient in this role than the antorbital cavity, which apparently held only an empty space as in modern archosaurs. Stem avians such as *Confuciusornis* probably had established their nasal cavity as an alternative device for brain cooling as the skull remodelling proceeded.

This hypothesis may provide another insight into the skull evolution. This transformation including a great reduction of the maxilla has been often discussed in relation with the tooth loss in theropods [84]. However, because teeth were lost several times independently in the stem clades of birds, the tooth loss alone was unlikely to be responsible for the reduction of the maxilla in the course of theropod evolution toward the modern birds [85]. The present study provides another reasonable cause for the maxilla reduction, that is, it was also driven by the requirement for an enlarged cross-section of the nasal cavity (figure 7).

## 5. Conclusion

The present study focused on the nasal structures with an aim of elucidating the primary role of the respiratory turbinate and the physiological function of the nasal cavity of non-avian dinosaurs. It was found that endotherms have a larger nasal cavity than ectotherms when regressed against the skull and head volumes. This result suggests the importance of a hitherto unheralded function of the respiratory turbinate, that is, more extensive thermal exchange for cooling large brains in birds and mammals. The size of the nasal cavity of the dromaeosaurid *V. mongoliensis* reconstructed based on inner cranial features was located below the regression line for extant endotherms, suggesting that most non-avialan theropod dinosaurs may not have possessed a fully developed nasal thermoregulation apparatus as modern birds do.

Based on the modification of the maxilla that constrains the form of the nasal cavity, a new hypothesis on the relationship between transformation of the skull and nasal cavity was proposed. It postulates that the great reduction of the maxilla on the theropod lineage resulted in the nasal cavity becoming an important apparatus for their thermal regulation strategy. The reduction and downward displacement of the maxilla after the origin of Avialae may indicate the timing of acquisition of the avian-like cephalic thermoregulation system.

## Data accessibility

The datasets supporting this article have been uploaded as part of the electronic supplementary material [86].
